# Supplementary material for: Integrated proteogenomic and metabolomic characterization of papillary thyroid cancer with different recurrence risks
Source: Nat Commun. 2024 Apr 12;15:3175. doi: 10.1038/s41467-024-47581-1 (PMC11014849; doi:10.1038/s41467-024-47581-1)
Supplement: Supplementary file 1 — Supplementary information [file 41467_2024_47581_MOESM1_ESM.pdf]

Integrated proteogenomic and metabolomic characterization of papillary thyroid cancer with different recurrence risks

Supplementary Information

Supplementary Figures

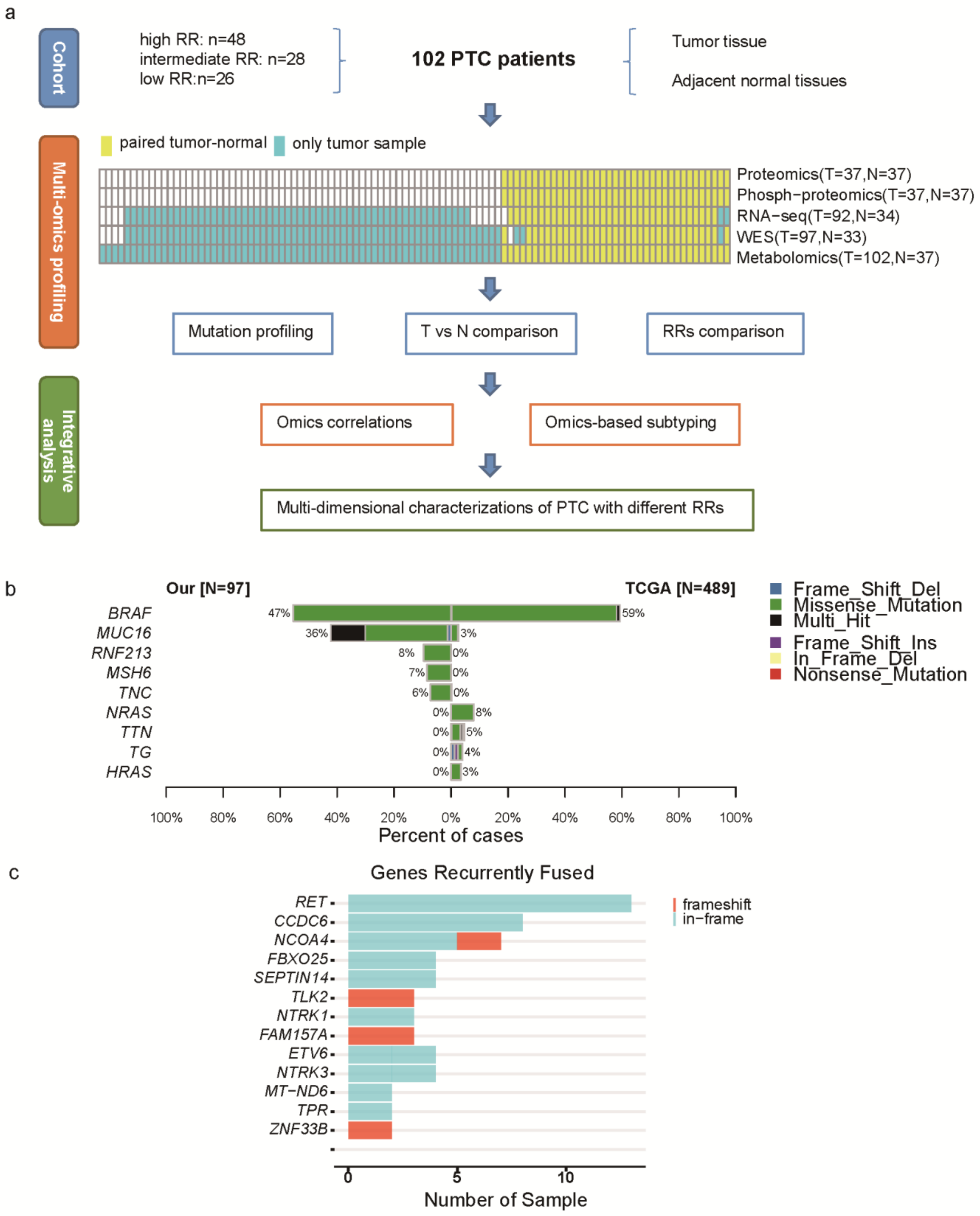

Fig S1. Genetic profile of the thyroid cancer patients with different recurrent risk.

a. Overview about the multi-omics study.

b. Comparison of the SMGs in our cohort and the TCGA-PTC dataset.

c. The genes recurrently fused based on the gene fusion results.

Source data are provided as a Source Data file.

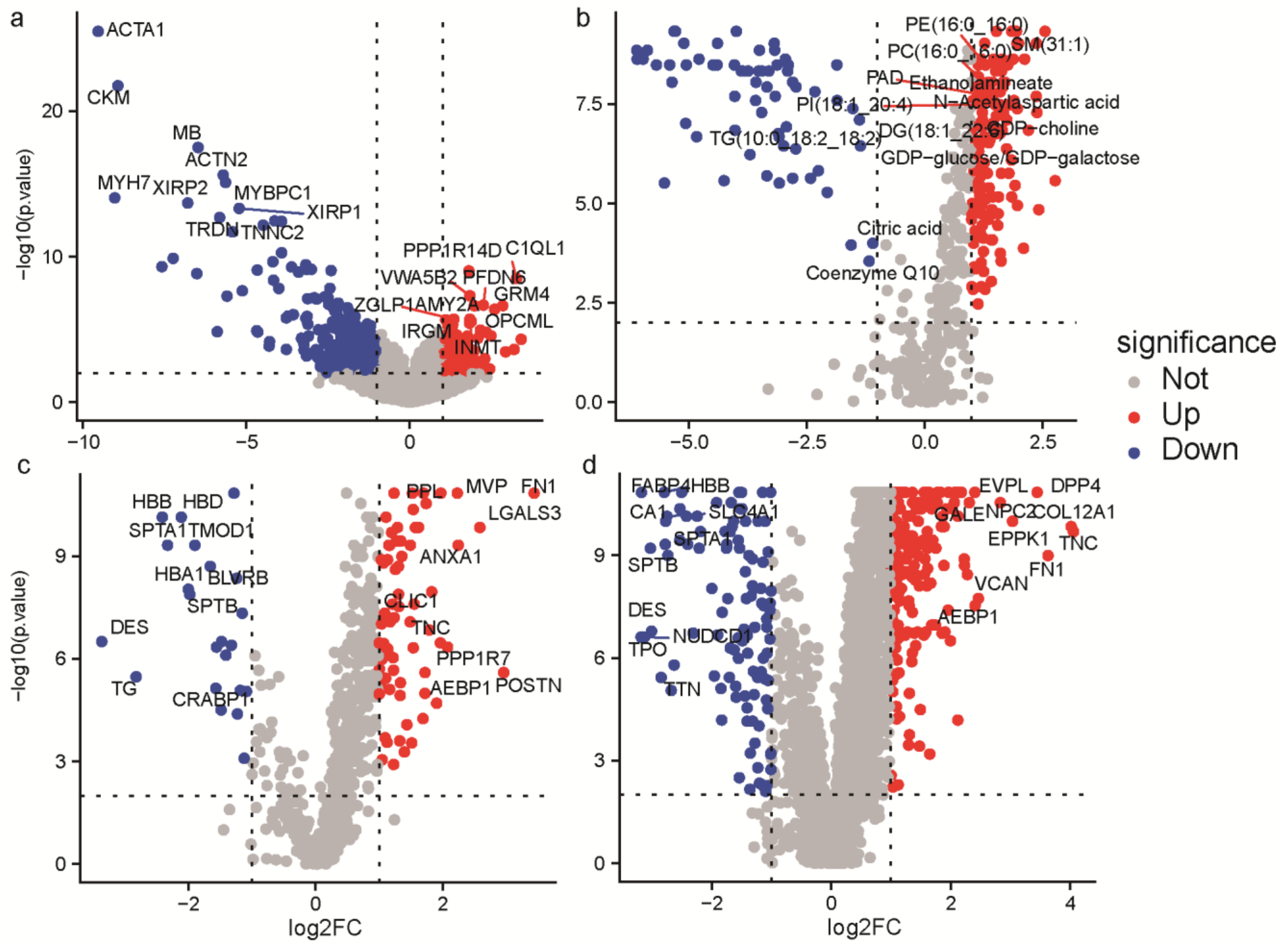

Fig S2. Differentially expressed molecules (DEMs) identified based on the multi-omics.

a. Differential expression analysis results for mRNA. DESeq2, two-sided.

b. Differential expression analysis results for metabolite. Wilcox-test, paired, two-sided.

c. Differential expression analysis results for Phospho protein. Wilcox-test, paired, two-sided.

d. Differential expression analysis results for protein. Wilcox-test, paired, two-sided.

Source data are provided as a Source Data file.

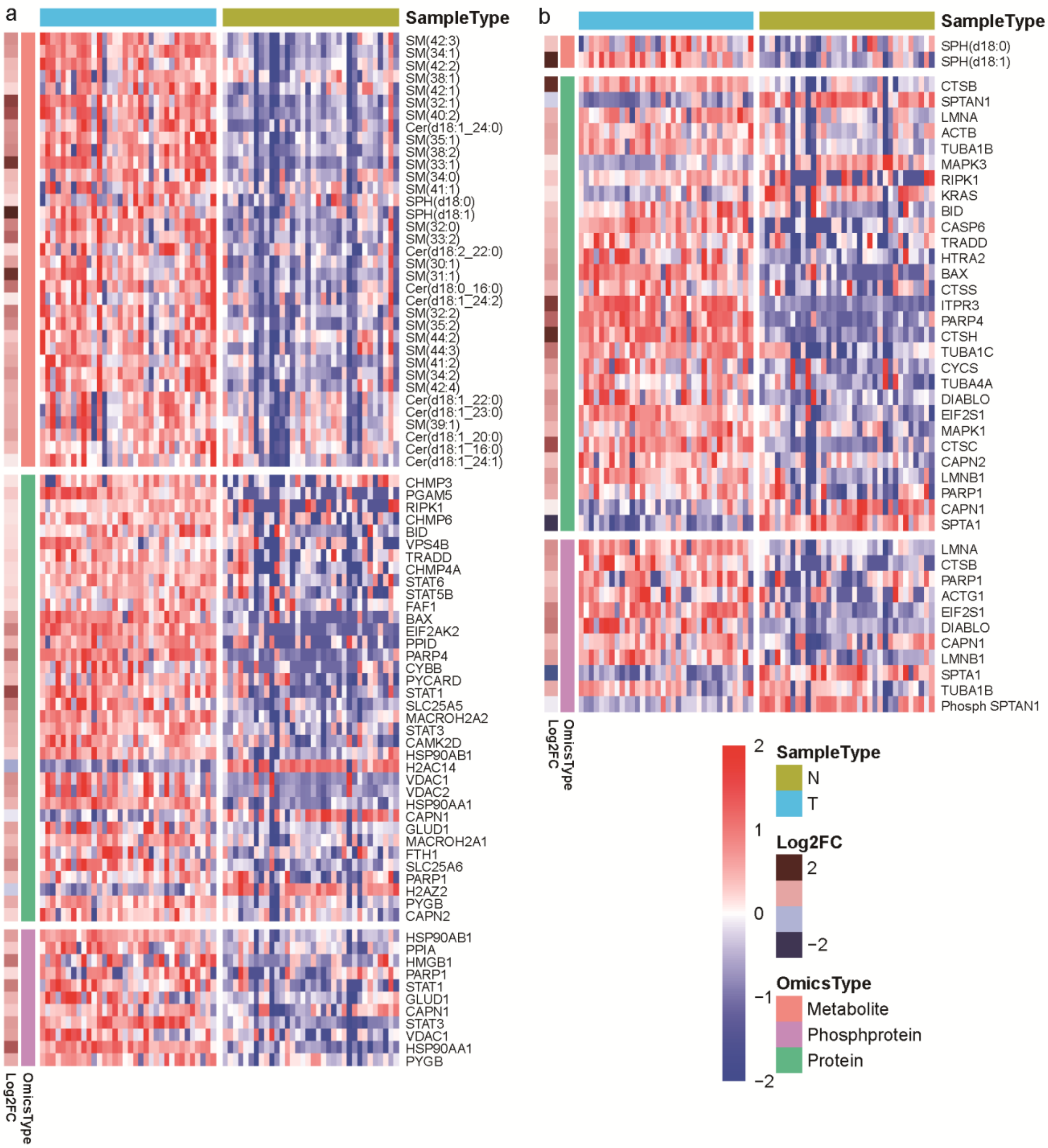

Fig S3. The expressional profiles of the differentially expressed molecules.

a-b. The expressional profiles of the differentially expressed molecules in necroptosis (a) and apoptosis (b) pathways (n=32 for both tumor and normal samples). Source data are provided as a Source Data file.

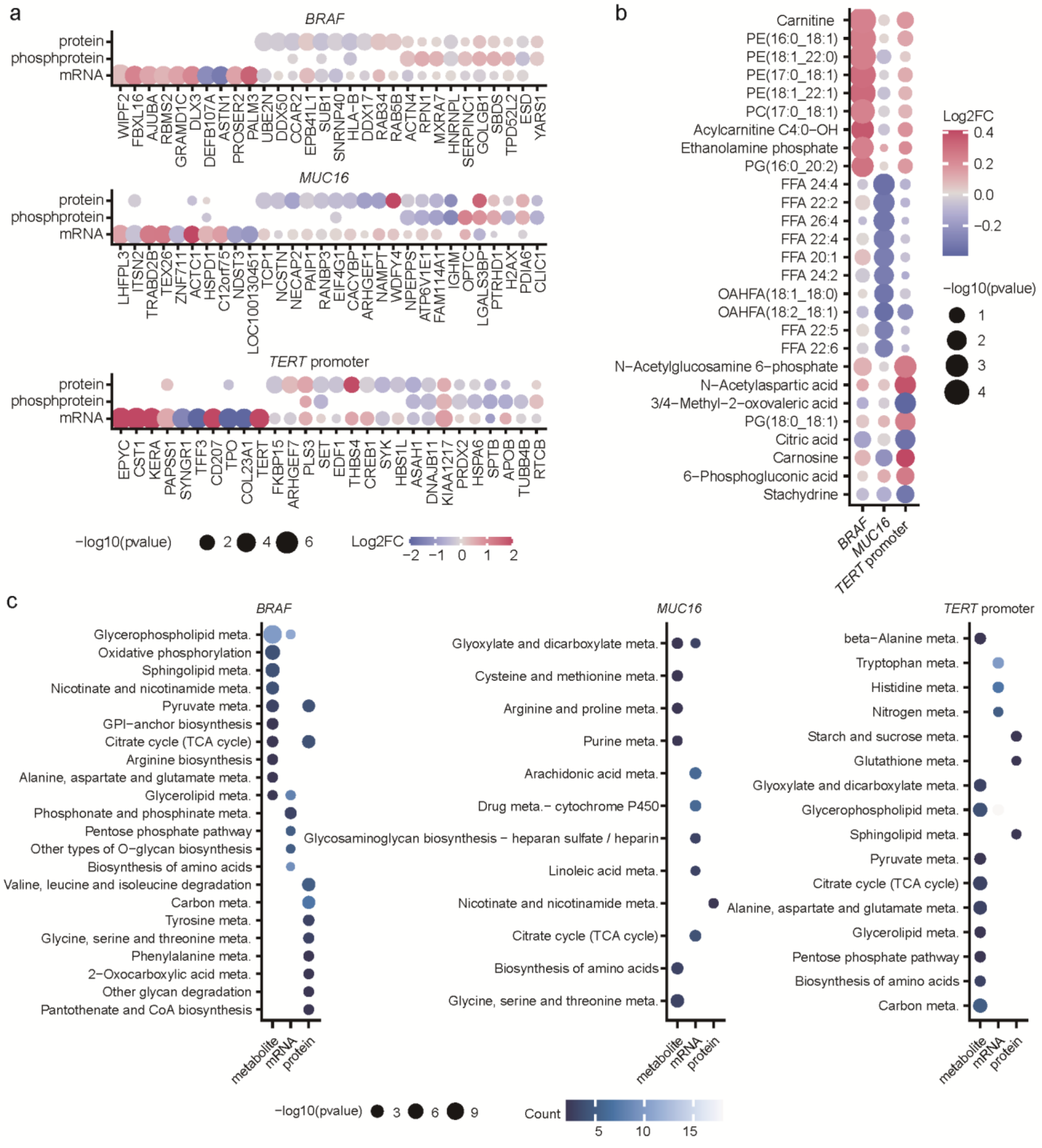

Fig S4. Multi-omics alterations related with gene mutations in *BRAF*, *MUC16* and *TERT* promoter.

a. Molecules with significant differential expressions in patients with and without mutations in *BRAF*, *MUC16* or *TERT* promoter (Wilcox-test, two-sided, P<0.05 and top-10 rank by Log2-FC in each type of omics for each mutation).

b. Metabolites with significant differential abundances in patients with and without mutations in *BRAF*, *MUC16* or *TERT* promoter (Wilcox-test, two-sided, P<0.05 and top-10 rank by Log2-FC for each mutation).

c. KEGG pathways enriched by the molecules with significant differential expressions (Wilcox-test, two-sided, P<0.05) in patients with and without mutations in *BRAF*, *MUC16* or *TERT* promoter (pathway enrichment examined by hypergeometric distribution, one-sided, P<0.05 and top-10 rank by P).

Source data are provided as a Source Data file.

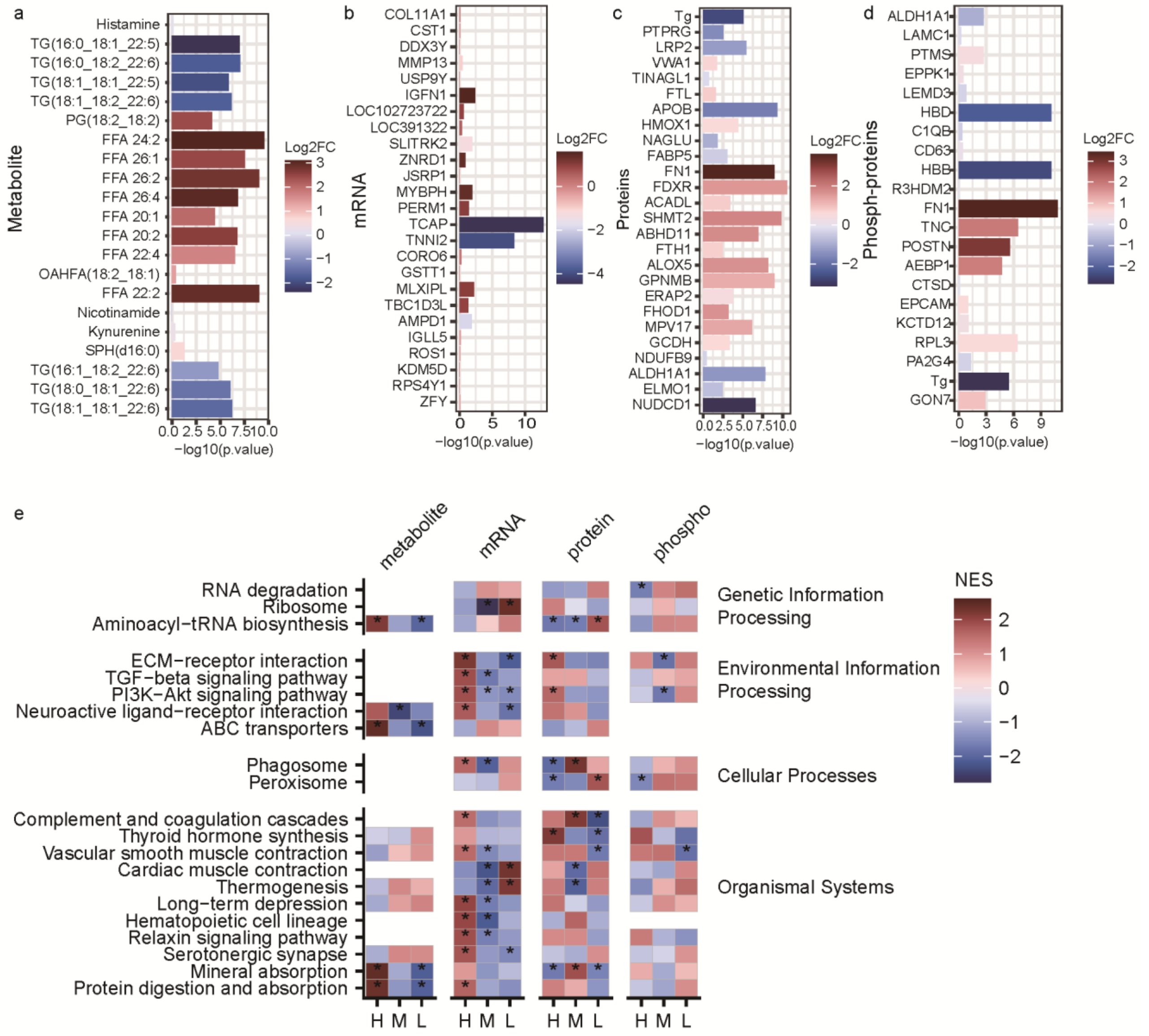

Fig S5. Recurrence risk relevant molecular and pathway characteristics.

a-d. The differential expressions between tumor and normal samples for the recurrence risk relevant metabolites (a), mRNAs (b), proteins (c) and phospho proteins (d). DESeq2 was applied to find the differentially expressed genes, and Wilcox-Test (paired, two sided) was applied to find the differentially expressed metabolites, proteins and phospho-proteins. e. The GSEA pathway enrichment results for PTC with different recurrence risks in addition to metabolism pathways. The results for metabolism pathways were included in Fig 3e. *: P<0.05, by Kolmogorov-Smirnov test, one-sided.

Source data are provided as a Source Data file.

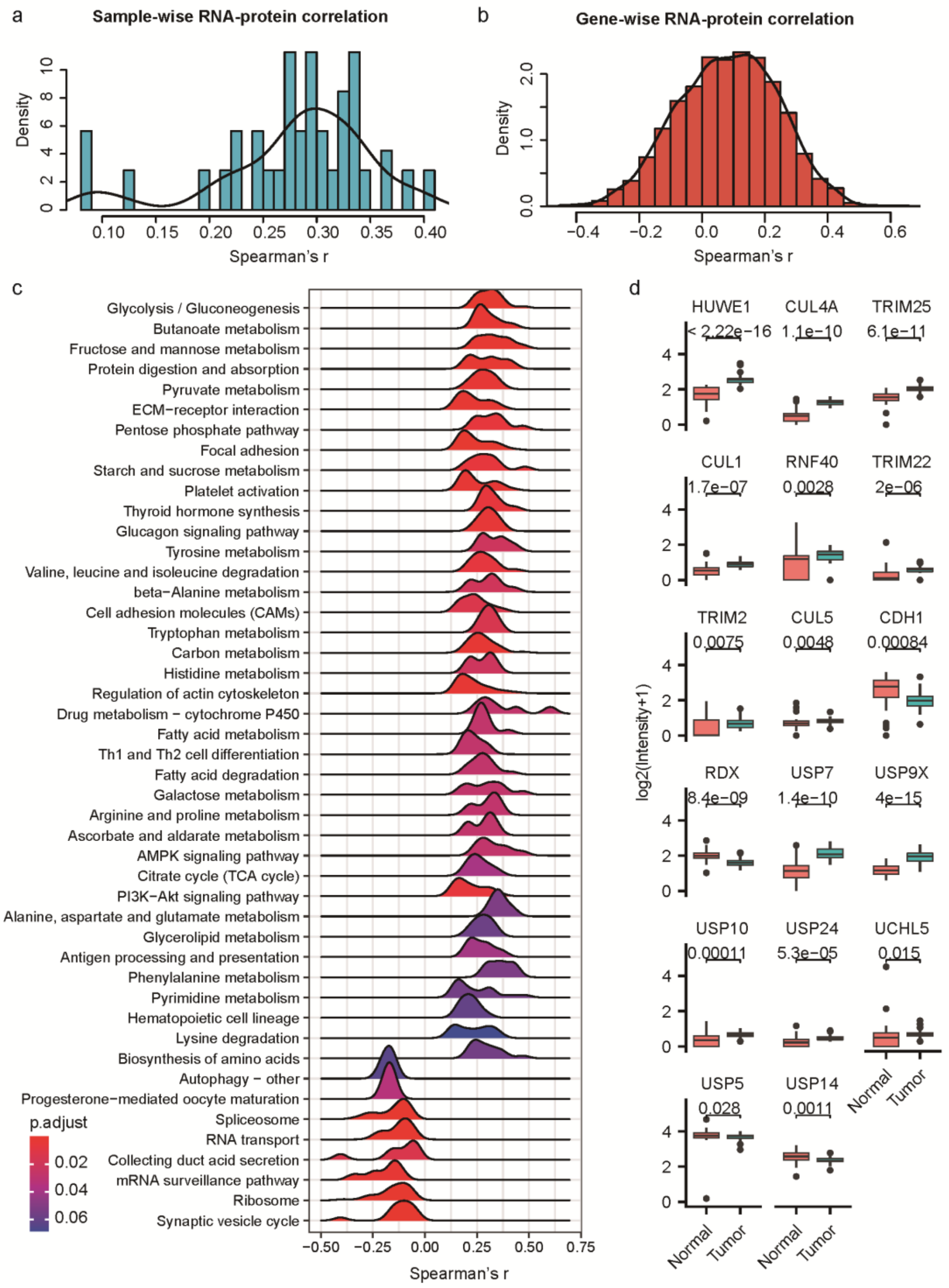

Fig S6. Low correlations between mRNAs and proteins.

a-b. Histogram of sample-wise (a) and gene-wise (b) mRNA-protein correlations. For the correlation analysis, only mRNAs and proteins that can be recognized by the same gene symbol names were considered, and only samples measured by both transcritomics and proteomics were used. For the sample-wise correlations, each individual sample was described by two vectors that respectively represeted the expressions of all available mRNAs and the corresponding proteins in this sample, and Spearman correlation coefficients between the two vectors were calculated. For the gene-wise correlations, each gene was described by two vectors that represented the expressions of this gene matched mRNA and protein across all the samples, and Spearman correlation coefficients between the two vectors were calculated.

c. KEGG pathways enriched for higher or low gene-wise mRNA-protein correlations (GSEA, P<0.05, Kolmogorov-Smirnov test, one-sided).

d. Boxplot of the protein expressions of E3 ubiquitin ligases or deubiquitinating enzymes in the normal (n=37) and tumor (n=37) samples. (Wicox-test, two-sided). In the boxplots, the central line represents median, the bounds of boxes represent the first and third quartiles, and the upper and lower whiskers extend to the highest or the smallest value within 1.5 interquartile range.

Source data are provided as a Source Data file.

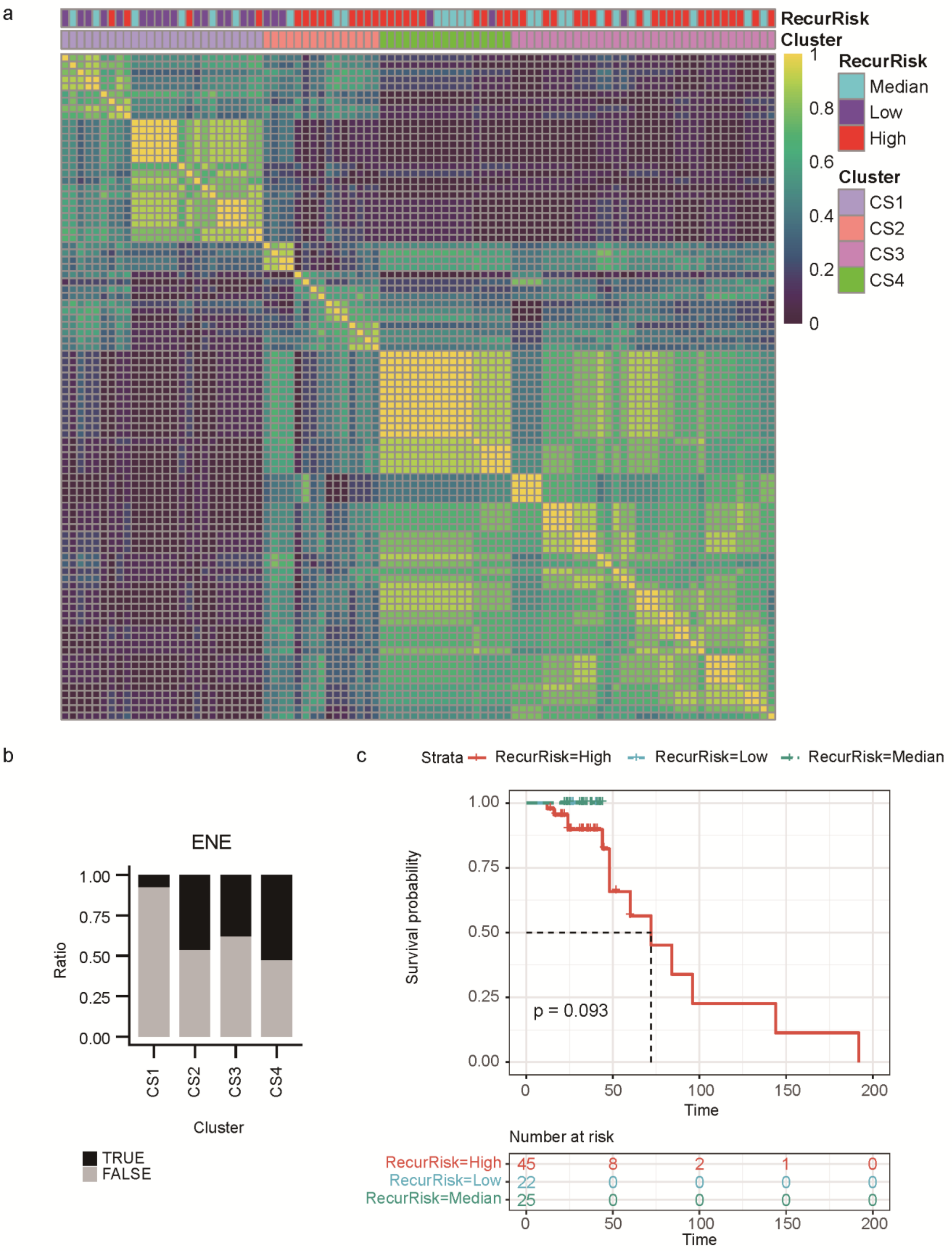

Fig S7. Clustering the patients based on the transcriptome and metabolome.

a. Heatmap of the sample similarity matrix.

b. The ratio of patients with and without ENE across the four subtypes.

c. KM-plot of the recurrence-free survival of patients stratified as high, intermediate and low recurrence risks (log-rank test, two-sided).

Source data are provided as a Source Data file.

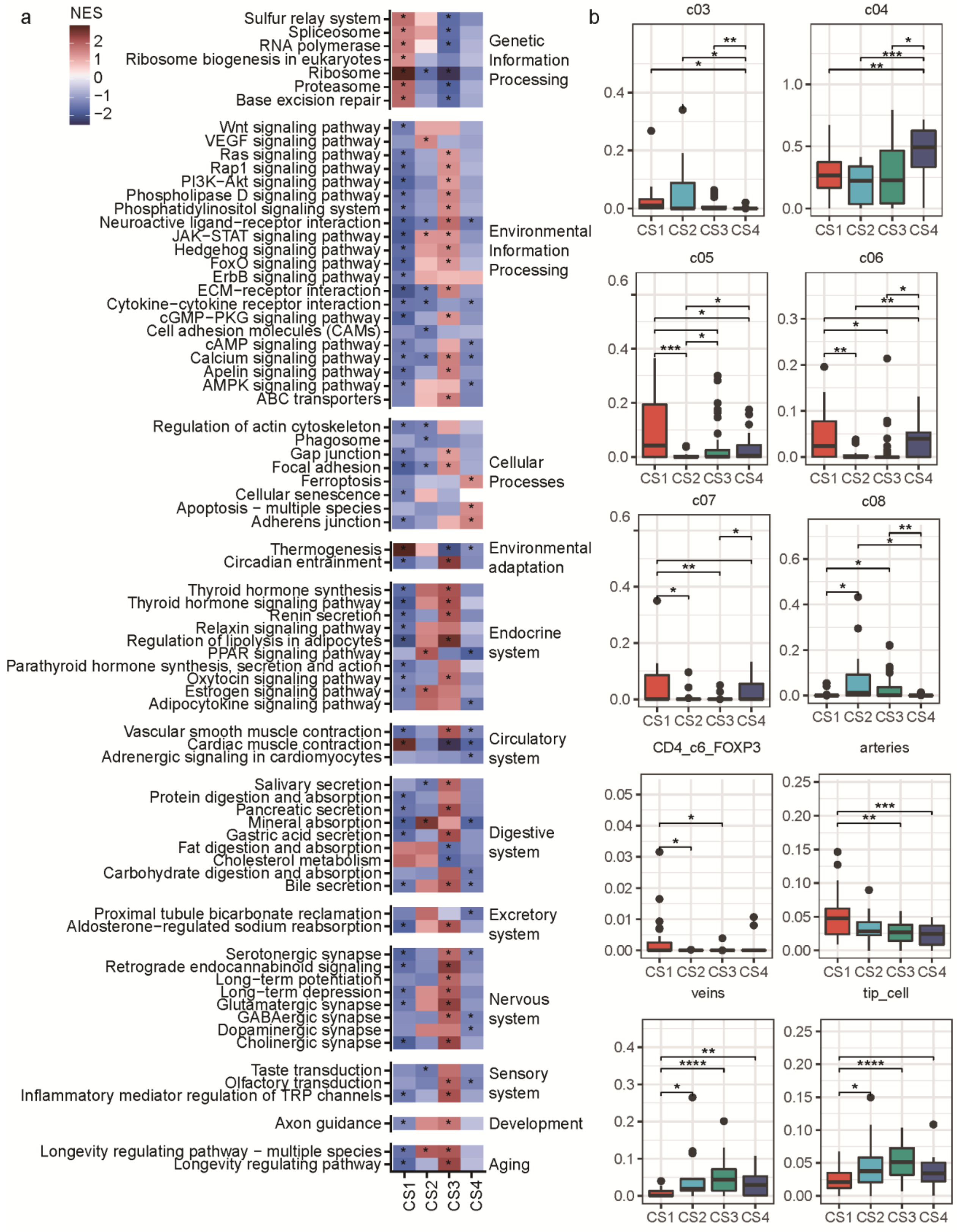

Fig S8. Pathway and cellular characterization of the four re-defined sub-types.

a. The GSEA pathway enrichment results for different sub-types based on transcriptomics data. The results for metabolism and immune pathways were included in Fig 6h-6i (Kolmogorov-Smirnov test, one-sided).

b. Box plot of the differential cell compositions of the four sub-types (Wilcox-Test, two-sided). In the boxplots, the central line represents median, the bounds of boxes represent the first and third quartiles, and the upper and lower whiskers extend to the highest or the smallest value within 1.5 interquartile range.

Source data are provided as a Source Data file.

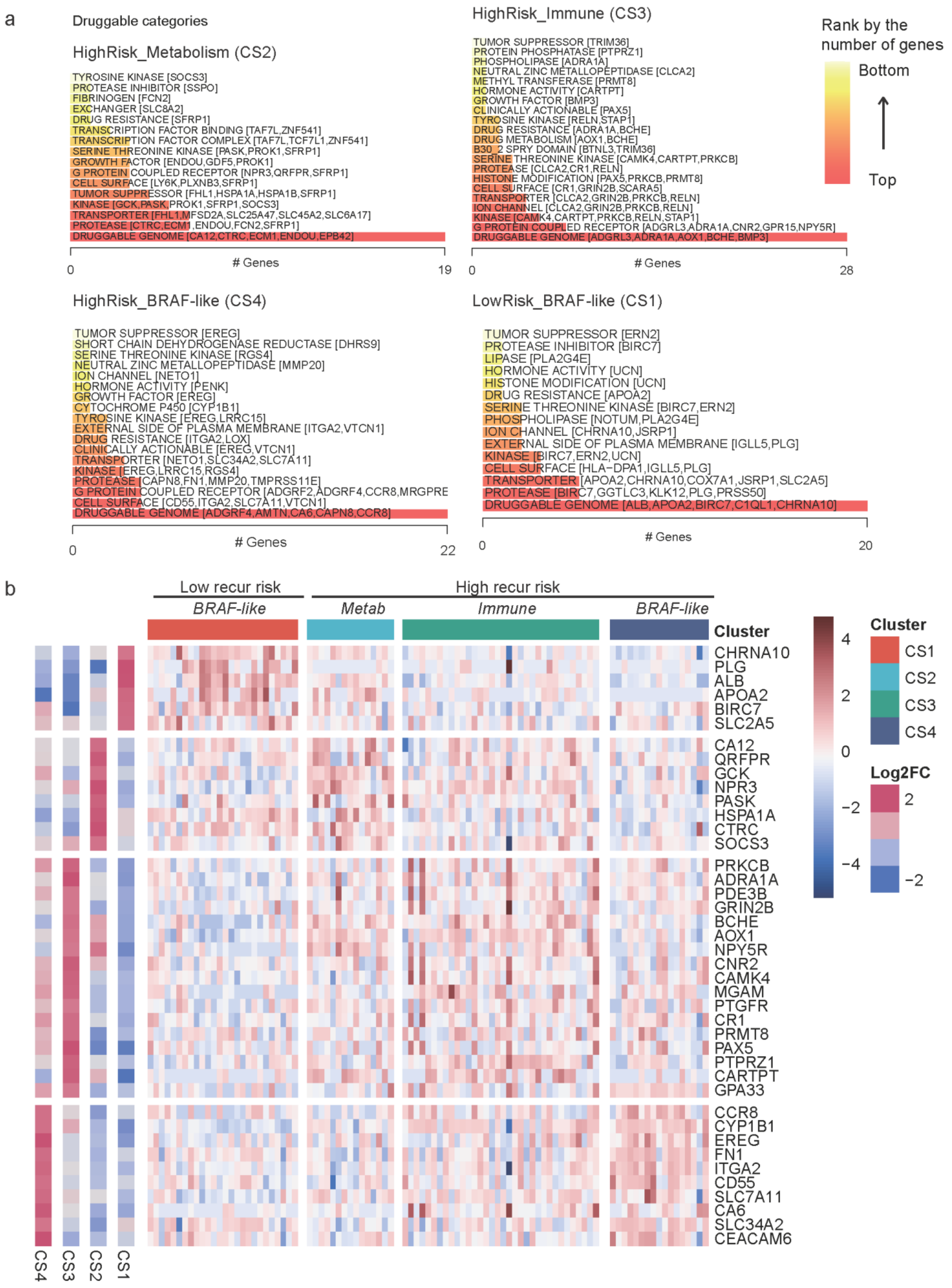

Fig S9. Potential targets of the four subtypes.

a. Druggable targets of the four subtypes. Druggable targets among the top-50 ranked subtype associated genes were selected based on the DGIdb database.

b. Heatmap of mRNA expression of the over-expressed druggable targets in the four clusters.

Source data are provided as a Source Data file.

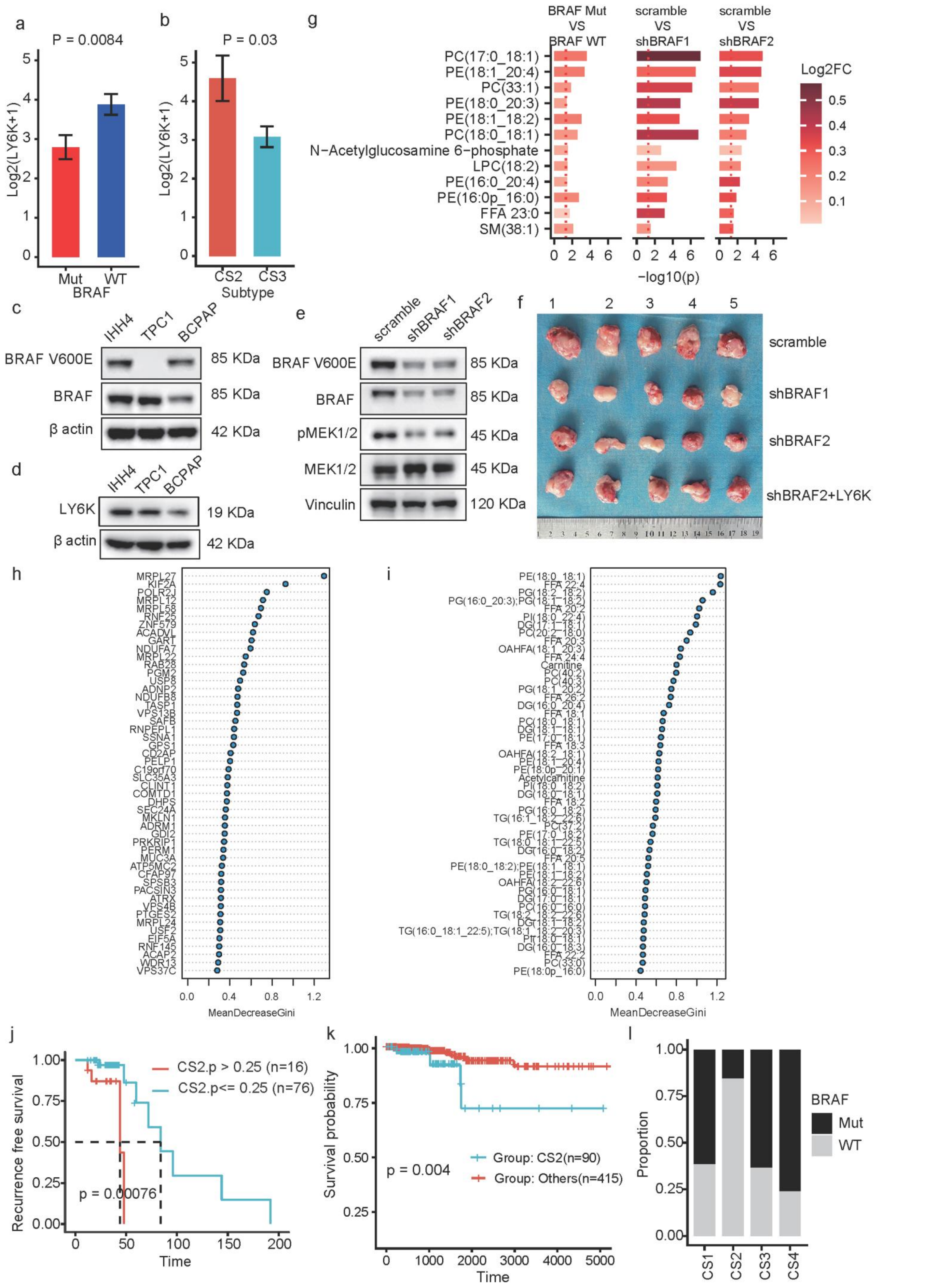

Figure S10. Validation of the subtypes by both experimental and computational methods

a. Bar plot of the mean expression of LY6K in PTC tumor tissues with mutant (Mut: n=46) and wild type (WT: n=45) BRAF. T-test, two-sided.

b. Bar plot of the mean expression of LY6K in the CS2 (n=15) and CS3 (n=34) subtypes. T-test, two-sided.
c-e. Immunoblotting analysis with indicated antibodies in IHH4, TPC1 and BCPAP cells. The experiments were repeated at least three times.
f. Diagram of excised tumors from indicated treatment IHH4 cells injected mice (Fig 7e, f) (*n*=5).
g. Bar plot of the differentially expressed metabolites between different conditions. The left-most column was based on the PTC tumor tissues and the other two columns were based on the cell lines;
h-i. Scatter plot showing the feature importance of the top-30 genes (g) and top-30 metabolites (h) for the subtype predictor. The MeanDecreaseGini was estimated based on the random forest algorithm.
j. KM-plot of the recurrence free survival curves of the PTC patients predicted with high and low CS2 probabilities (threshold was set as 0.25, since there were four subtypes). P: Log-rank test.
k. KM-plot of the over all survival curves of the predicted CS2 and other subtypes in the TCGA-PTC dataset. P: Log-rank test.
l. Bar plot of the proportion of BRAF statuses across the four predicted clusters in the TCGA-PTC dataset.
Source data are provided as a Source Data file.
