# Supplementary material for: Integrated proteogenomic and metabolomic characterization of papillary thyroid cancer with different recurrence risks
Source: Nat Commun. 2024 Apr 12;15:3175. doi: 10.1038/s41467-024-47581-1 (PMC11014849; doi:10.1038/s41467-024-47581-1)
Supplement: Supplementary file 3 — Description of Additional Supplementary Files [file 41467_2024_47581_MOESM3_ESM.pdf]

Description of Additional Supplementary Files

File Name: Supplementary Data 1.

Description: The clinicopathological characteristics of the PTC samples.

File Name: Supplementary Data 2.

Description: The GSEA results based on the gene-wise mRNA-protein correlation coefficients. P: Kolmogorov-Smirnov test, one-sided .
