# Supplementary material for: Integrated proteogenomic and metabolomic characterization of papillary thyroid cancer with different recurrence risks
Source: Nat Commun. 2024 Apr 12;15:3175. doi: 10.1038/s41467-024-47581-1 (PMC11014849; doi:10.1038/s41467-024-47581-1)
Supplement: Supplementary file 6 — Reporting Summary [file 41467_2024_47581_MOESM6_ESM.pdf]

nature portfolio

Corresponding author(s): Hai-long Piao

Last updated by author(s): Feb 20, 2024

Reporting Summary

Nature Portfolio wishes to improve the reproducibility of the work that we publish. This form provides structure for consistency and transparency in reporting. For further information on Nature Portfolio policies, see our Editorial Policies and the Editorial Policy Checklist.

Statistics

For all statistical analyses, confirm that the following items are present in the figure legend, table legend, main text, or Methods section.

n/a	Confirmed	
☐	☒	The exact sample size (*n*) for each experimental group/condition, given as a discrete number and unit of measurement
☐	☒	A statement on whether measurements were taken from distinct samples or whether the same sample was measured repeatedly
☐	☒	The statistical test(s) used AND whether they are one- or two-sided<br>*Only common tests should be described solely by name; describe more complex techniques in the Methods section.*
☐	☒	A description of all covariates tested
☐	☒	A description of any assumptions or corrections, such as tests of normality and adjustment for multiple comparisons
☐	☒	A full description of the statistical parameters including central tendency (e.g. means) or other basic estimates (e.g. regression coefficient) AND variation (e.g. standard deviation) or associated estimates of uncertainty (e.g. confidence intervals)
☐	☒	For null hypothesis testing, the test statistic (e.g. *F*, *t*, *r*) with confidence intervals, effect sizes, degrees of freedom and *P* value noted<br>*Give P values as exact values whenever suitable.*
☒	☐	For Bayesian analysis, information on the choice of priors and Markov chain Monte Carlo settings
☒	☐	For hierarchical and complex designs, identification of the appropriate level for tests and full reporting of outcomes
☐	☒	Estimates of effect sizes (e.g. Cohen's *d*, Pearson's *r*), indicating how they were calculated

Our web collection on statistics for biologists contains articles on many of the points above.

Software and code

Policy information about availability of computer code

Data collection	No software was used to do data collection.
Data analysis	Data analysis was done by R (v 3.6.3 and v 4.0.4) and mixOmics (v 6.10.9), DWLS (v 0.1.0), MOVICS (v 0.99.17), annoFuse (V 0.90.0) , DESeq2(v 1.26.0) , clusterProfiler (v 3.18.1), survival (v 3.2-7) and survminer (v0.4.9) packages and the codes can be found at : https://github.com/ diChen310/PTC_multi_omics

For manuscripts utilizing custom algorithms or software that are central to the research but not yet described in published literature, software must be made available to editors and reviewers. We strongly encourage code deposition in a community repository (e.g. GitHub). See the Nature Portfolio guidelines for submitting code & software for further information.

Data

Policy information about availability of data

All manuscripts must include a data availability statement. This statement should provide the following information, where applicable:

- Accession codes, unique identifiers, or web links for publicly available datasets
- A description of any restrictions on data availability
- For clinical datasets or third party data, please ensure that the statement adheres to our policy

The raw WES and RNA-seq data of the PTC samples have been deposited in the Genome Sequence Archive in National Genomics Data Center , China National Center for Bioinformation / Beijing Institute of Genomics, Chinese Academy of Sciences (GSA-Human) under accession code HRA005293 (https://ngdc.cncb.ac.cn/

gsa-human/browse/HRA005293) and HRA005382 (https://ngdc.cncb.ac.cn/gsa-human/browse/HRA005382). The raw WES and RNA-seq data are available under restricted access for research purposes only, access can be obtained by the DAC (Data Access Committees) of the GSA-human database. According to the guidelines of GSA-human, all non-profit researchers can obtain access to the data, and the Principle Investigator of any research group is allowed to apply the data. The access authority can be obtained for Research Use Only. The user can also contact the corresponding author directly. Once access has been approved, the data will be available to download for 2 months. The mass spectrometry proteomics and phospho-proteomics data have been deposited to the ProteomeXchange Consortium via the PRIDE with the dataset identifier PXD044900 (https://www.ebi.ac.uk/pride/archive/projects/PXD044900) and PXD045017 (https://www.ebi.ac.uk/pride/archive/projects/PXD045017). The metabolomics data have been deposited to MetaboLights [70] (www.ebi.ac.uk/metabolights/MTBLS3339). Transcriptomics and survival data of TCGA-PTC samples were obtained from Genomic Data Commons (https://portal.gdc.cancer.gov/projects/TCGA-THCA). The scRNA-seq data used in this study are available in the Gene Expression Omnibus repository under accession code GSE184362 (https://www.ncbi.nlm.nih.gov/geo/query/acc.cgi?acc=GSE184362). Source data are provided with this paper.

Research involving human participants, their data, or biological material

Policy information about studies with human participants or human data. See also policy information about sex, gender (identity/presentation), and sexual orientation and race, ethnicity and racism.

Reporting on sex and gender	Both male and female samples were included. The gender information was described in Table S1. This study is mainly focused on the multi-omics based molecular features of recurrent papillary thyroid cancer and exploration of new subtypes in general, regardless of the gender. None of the conclusions were specific to one gender, so no gender-specific analysis was performed.
Reporting on race, ethnicity, or other socially relevant groupings	All the samples were from Chinese people, and the other socially relevant grouping information were not involved in this study.
Population characteristics	The population characteristics are described in Table 1 and Supplementary Data 1.
Recruitment	The sample collection, store and quality control were in accordance with the standard operation procedures of the Institutional Tissue Bank (ITB) of FUSCC. As described in the previous study [23], after the samples were detached from the human body, they were stored in liquid nitrogen within 30 minutes, and they were made into frozen sections and paraffin-embedded sections at the same time, which were then stained by hematoxylin and eosin. All hematoxylin and eosin slides of the samples were subjected to evaluation for histopathological morphology and tumor components by expert pathologists. The samples enrolled in this study should meet the following criteria: (1) the percentage of tumor cell nuclear (tumor cell nuclear/total cell nuclear)≥80%??2?the percentage of total cells≥80% (cell area/ total tissue section area) and (3) the percentage of necrosis≤20% (necrotic tissue area/total tissue section area).
Ethics oversight	The study is approved by the Ethical Committee of the Fudan University Shanghai Cancer Center.Each patient provided a written informed consent for his/her specimens and information to be used for research and stored in the hospital database, and this study was approved by the Ethical Committee of the FUSCC. All procedures performed in our study were in accordance with the ethical standards of our institutional research committee and with the 1964 Helsinki declaration and its later amendments or comparable ethical standards.

Note that full information on the approval of the study protocol must also be provided in the manuscript.

Field-specific reporting

Please select the one below that is the best fit for your research. If you are not sure, read the appropriate sections before making your selection.

☒ Life sciences ☐ Behavioural & social sciences ☐ Ecological, evolutionary & environmental sciences

For a reference copy of the document with all sections, see nature.com/documents/nr-reporting-summary-flat.pdf

Life sciences study design

All studies must disclose on these points even when the disclosure is negative.

Sample size	A total of 102 pillary thyroid cancer was collected. The sample size was determined by the number of clinical available samples where the sample collection, store and quality control were in accordance with the standard operation procedures of the Institutional Tissue Bank (ITB) of the Fudan University Shanghai Cancer Center. For the experiments, when used in a power calculation, our sample size predetermination experiments indicate that 5 mice per group can identify the tumour size and weight (P<0.05 with 100% power).
Data exclusions	Samples did not meet the following criteria: (1) the percentage of tumor cell nuclear (tumor cell nuclear/total cell nuclear)≥80%??2?the percentage of total cells≥80% (cell area/ total tissue section area) and (3) the percentage of necrosis≤20% (necrotic tissue area/total tissue section area) were excluded from this study.
Replication	All analysis and statistics were reproducible. The experiments were repeated at least three times.
Randomization	Random sampling was done in R based on the createDataPartition function of the caret (v 6.0-88) package to randomly split the PTC data into training and testing datasets. Animals were randomly assigned to different groups.

Blinding	No blinding was done since the group information like the recurrence risk types are important for the computation of the study.

Reporting for specific materials, systems and methods

We require information from authors about some types of materials, experimental systems and methods used in many studies. Here, indicate whether each material, system or method listed is relevant to your study. If you are not sure if a list item applies to your research, read the appropriate section before selecting a response.

Materials & experimental systems

n/a	Involved in the study
☐	☒ Antibodies
☐	☒ Eukaryotic cell lines
☒	☐ Palaeontology and archaeology
☐	☒ Animals and other organisms
☒	☐ Clinical data
☒	☐ Dual use research of concern
☒	☐ Plants

Methods

n/a	Involved in the study
☒	☐ ChIP-seq
☒	☐ Flow cytometry
☒	☐ MRI-based neuroimaging

Antibodies

Antibodies used	Primary antibodies against BRAFV600E was purchased from Abcam(Catalog:ab228461, dilution 1:500), and BRAF was from Santa Cruz Biotechnology(Catalog:sc-5284, dilution 1:1000). Primary antibodies against LY6K was purchased from Beyotime Biotechnology (Catalog:AG5061, dilution 1:1000), beta- actin and vinculin were purchased from Proteintech (Catalog: 81115-1-RR, dilution 1:2000) and Santa Cruz Biotechnology (Catalog:sc-73614, dilution 1:2000), respectively. Primary antibodies pMEK1/2 (ser 217/221) and MEK1/2 were purchased from Cell Signaling Technology (Catalog: 8727T, dilution 1:2000 and Catalog: 9154T, dilution 1:1000).
Validation	Antibodies were validated by the manufacturers as stated on their websites. described on the following websites: Mouse anti-BRAFV600E(ab228461, Abcam[illegible][https://www.abcam.com/products/primary-antibodies/braf-mutated-v600e-antibody-ve1-ab228461.html], Mouse anti-BRAF(sc-5284;Santa Cruz Biotechnology)[https://www.scbt.com/p/raf-b-antibody-f-7], Rabbit anti-LY6K(AG5061;Beyotime Biotechnology)[https://www.beyotime.com/product/AG5061.htm], Mouse anti-Actin (81115-1-RR;Proteintech Group)[https://www.ptglab.com/products/beta-actin-Antibody-81115-1-RR.htm], MEK1/2 (D1A5) Rabbit mAb [https://www.cellsignal.cn/products/primary-antibodies/mek1-2-d1a5-rabbit-mab/8727], Phospho-MEK1/2 (Ser217/221) (41G9) Rabbit mAb [https://www.cellsignal.cn/products/primary-antibodies/phospho-mek1-2-ser217-221-41g9-rabbit-mab/9154]

Eukaryotic cell lines

Policy information about cell lines and Sex and Gender in Research

Cell line source(s)	Human IHH4(sex: male) was purchased from JCRB Cell Bank<br>Human TPC1(sex: female) was purchased from Cell Bank(Chinese Academy of Sciences)<br>Human BCPAP(sex: female) was purchased from Cell Bank(Chinese Academy of Sciences)<br>Human HEK293T(sex: female) was purchased from ATCC
Authentication	None of the cell lines was authenticated.
Mycoplasma contamination	Cell lines were tested routinely and were all negative for mycoplasma contamination.
Commonly misidentified lines (See ICLAC register)	None of the misidentified lines were used.

Animals and other research organisms

Policy information about studies involving animals; ARRIVE guidelines recommended for reporting animal research, and Sex and Gender in Research

Laboratory animals	Mice were bred in our animal facilities under specific pathogen-free conditions in a temperature-controlled environment?which maintained at 25? with 50% humidity and a 12/12-hour light-dark cycle.
Wild animals	We did not use any wild animals.
Reporting on sex	Only male mice were utilized
Field-collected samples	This study did not involve any field-collected samples

Ethics oversight	All experiments were carried out according to the regulations set by the Ethics Committee of China Medical University.

Note that full information on the approval of the study protocol must also be provided in the manuscript.
